# Use of Seasonal Influenza Vaccination and Its Associated Factors among Elderly People with Disabilities in Taiwan: A Population-Based Study

**DOI:** 10.1371/journal.pone.0158075

**Published:** 2016-06-23

**Authors:** Yu-Chia Chang, Ho-Jui Tung, Shang-Wei Hsu, Lei-Shin Chen, Pei-Tseng Kung, Kuang-Hua Huang, Shang-Jyh Chiou, Wen-Chen Tsai

**Affiliations:** 1 Department of Healthcare Administration, Asia University, Taichung, Taiwan, ROC; 2 Department of Medical Research, China Medical University Hospital, China Medical University, Taichung, Taiwan, ROC; 3 Department of Health and Kinesiology, Texas A&M University, College Station, Texas, United States of America; 4 Department of Health Services Administration, China Medical University, Taichung, Taiwan, ROC; 5 Department of Health Care Management, National Taipei University of Nursing and Health Sciences, Taipei, Taiwan, ROC; Qom University, ISLAMIC REPUBLIC OF IRAN

## Abstract

**Background:**

Influenza immunization among elderly people with disabilities is a critical public health concern; however, few studies have examined the factors associated with vaccination rates in non-Western societies.

**Methods:**

By linking the National Disability Registration System and health service claims dataset from the National Health Insurance program, this population-based study investigated the seasonal influenza vaccination rate among elderly people with disabilities in Taiwan (N = 283,172) in 2008. A multivariate logistic regression analysis was conducted to adjust for covariates.

**Results:**

Nationally, only 32.7% of Taiwanese elderly people with disabilities received influenza vaccination. The strongest predictor for getting vaccinated among older Taiwanese people with disabilities was their experience of receiving an influenza vaccination in the previous year (adjusted odds ratio [AOR] = 6.80, 95% confidence interval [CI]: 6.67–6.93). Frequent OPD use (AOR = 1.85, 95% CI: 1.81–1.89) and undergoing health examinations in the previous year (AOR = 1.66, 95% CI: 1.62–1.69) also showed a moderate and significant association with receiving an influenza vaccination.

**Conclusions:**

Although free influenza vaccination has been provided in Taiwan since 2001, influenza immunization rates among elderly people with disabilities remain low. Policy initiatives are required to address the identified factors for improving influenza immunization rates among elderly people with disabilities.

## Introduction

Seasonal influenza, a critical public health concern, has caused substantial disease-related and economic burdens worldwide. Globally, the annual epidemics of seasonal influenza have been estimated to contribute to 3–5 million severe cases and approximately 250,000–500,000 deaths [1]. Approximately 90% of seasonal influenza-related deaths and 50%–70% of seasonal influenza-related hospitalizations occur among elderly aged 65 years or older and high risk groups [2, 3]. Although increasing evidence demonstrates that available influenza vaccines are less effective in the elderly compared to younger adults, vaccination is still considered the most efficacious methods currently available of preventing influenza and its severe complications, particularly in immunocompromised elderly or those with high-risk medical conditions [4–7].

The World Report on Disability has estimated that more than a billion people are living with some form of disability. As the global population ages, the number of people with disabilities is expected to grow rapidly [8]. In Taiwan, according to the statistics of the Taiwanese registration system for people with disabilities (maintained by the Ministry of Health and Welfare), the percentage of people with disabilities in Taiwan reached 2.30% of the total population in 1997. By 2014, it had increased to 4.87% of the total population, and 38.18% of them were adults aged 65 years and older [9]. The pace of population aging in Taiwan is one of the rapidest in the world and, thus, both primary prevention and health promotion among the disabled elderly are urgently needed [10].

Studies have reported that older people with disabilities may have poorer health status and consequently, a higher demand for health care than their counterparts without disabilities [8, 11, 12], but they are more likely to encounter difficulties in accessing health care services in spite of this [12–17]. Since prevention is better than cure, primary preventions, such as seasonal influenza vaccination, should be placed in higher priority for the elderly with disabilities. If the elderly with disabilities contract influenza, their poorer medical conditions will make them more vulnerable to influenza’s severe complications. Plus, in addition to the direct medical costs of treating influenza, extra or auxiliary care resources would be required from their lay caregivers or family members. Thus, elderly people with disabilities should be the target population for immunization against seasonal influenza [5, 8, 17, 18].

Although influenza vaccination among elderly people with disabilities is a critical public health concern, the rates and factors associated with receiving influenza vaccination among people with disabilities have not been thoroughly studied. There are only few studies that explored this topic, all of them were published in the United States, and the results were inconclusive [17, 19–22]. Furthermore, most of these studies used self-reported disability and influenza vaccination status from survey databases; therefore, they were subject to recall biases. In Taiwan, free influenza vaccination has been provided to people aged 65 years and older since 2001. To further understand the rates of seasonal influenza vaccination use among elderly people with disabilities in an Asian context with a universal health care insurance system, we used a nationwide, population-based data set to investigate the use of influenza vaccination among elderly people with disabilities in Taiwan and its associated factors.

## Materials and Methods

### Background information

In Taiwan, a universal insurance scheme called the National Health Insurance (NHI) was launched in 1995; which is a single-payer national health insurance plan that covers more than 99% of the citizens of Taiwan [23, 24]. The policy of free influenza vaccination for the elderly has been implemented through the NHI to people aged 65 years and older since 2001. Each year, starting from October 1, enrolled seniors can have a visit to all NHI-licensed clinics or hospitals to receive free influenza vaccination [25].

### Data source and processing

The subjects in this study were derived from the National Disability Registration System, in which all disabled residents must register for claiming disability benefits. People aged 65 years and older in 2008 were selected from the system and linked to their health service records managed by the Bureau of National Health Insurance (NHI), which covers over 99% of the population of Taiwan. The Statistics Center of the Ministry of Health and Welfare linked the two databases. Data related to the identification of individuals were encrypted before being released to the researchers, and personal privacy was therefore protected. This study was approved by the Research Ethics Committee of China Medical University and Hospital (IRB No. CMU-REC-101-012).

Among 25 cities (or counties) in Taiwan, medical claims data from Kaohsiung City, Kaohsiung County, and Taoyuan County were not available at the time of linking the databases. The number of people with disabilities in these three areas accounted for 18.2% of the total disabled population in Taiwan. Thus, over 80% of the elderly people with disabilities in Taiwan were included in the current analysis. Therefore, the study sample can be considered highly representative, and minimal variations were observed in the data structure [26].

### Study variables

The predictors included in this study for evaluating the factors associated with seasonal influenza vaccination can be divided into the following components: (a) The levels and types of disabilities obtained from the National Disability Registration System. Disability types were physical, mental, and both. The Physically and Mentally Disabled Citizens Protection Act in Taiwan classifies disabilities into 17 categories [27]. In this study, physical disabilities included visual impairment, hearing impairment, sound and speech impairment, physical disability, multiple disabilities, major organ malfunction, facial injury, balance impairment, refractory epilepsy, and rare diseases. Mental disabilities included intellectual impairment, dementia, autism, chromosomal abnormalities, metabolic abnormalities, congenital defects, and psychiatric disorders. All types of disabilities were further classified into four levels: extremely severe, severe, moderate, and mild. (b) Demographic characteristics, which included gender, age, education level, marital status, aboriginal status, and premium-based monthly salary. (c) Health status, which included Charlson Comorbidity Index (CCI) severity. (d) The health care utilization records during the preceding year, which were derived from the claims data and contain the number of outpatient department (OPD) visits for all causes, any hospitalizations for all causes, influenza vaccination, and any health examination. The level of medical facilities (i.e., medical center, regional hospital, district hospital, and clinic) where the subjects received their health services most frequently were also included as a predictor, as was the type of ownership of the medical facilities.

The dependent variable, whether or not a study subject received influenza vaccination, was determined by examining the medical claims data between October 1, 2008 and December 31, 2008, when the seasonal influenza vaccines are freely available every year to people aged 65 years and older in Taiwan.

### Statistical analysis

We first used a descriptive analysis to examine the frequency and percentage of elderly people with disabilities receiving influenza vaccination in 2008 and the characteristics of the study variables. Both the chi-square test and *t*-test were used to compare differences between receiving influenza vaccination and each variable and to analyze the statistical significance. Furthermore, logistic regression models were used to examine the factors associated with the use of influenza vaccination in 2008 among elderly people with disabilities. The odds ratios (ORs) and 95% confidence intervals (CIs) were calculated for each model. All statistical analyses were conducted using SAS version 9.3 (SAS institute, Inc., Cary, NC, USA). Statistical significance was defined at the *p* < .05 level.

## Results

The details of the study sample are presented in Table 1. A total of 283,172 elderly people with disabilities qualified for the free influenza vaccination in 2008. The influenza vaccination rate was 32.70%. Of all the study subjects, 89.75% had physical disabilities, 8.11% had mental disabilities, and 2.14% had both disabilities. Regarding the severity of disabilities, most of the subjects had mild (34.29%) and moderate (32.21%) disabilities. The average age of the sample was 77.07 years, 51.97% of them were male, and 69.74% were in the “elementary or illiterate” category of educational level. Most of the elderly people with disabilities were married (62.96%), and only 1.43% of the subjects had aboriginal status. As to the distribution of premium-based monthly salary, most of the subjects belonged to the “≤ NT$22,800 (New Taiwan Dollars)” category. Approximately 31.07% of the elderly people with disabilities did not have any comorbidities. In terms of the health care utilization measures in the preceding year (2007), 33.48% of the subjects were hospitalized, 24.99% underwent health examinations, and only 29.59% received influenza vaccination. All of these factors significantly correlated with receiving seasonal influenza vaccination in 2008 (Table 1).

**Table 1:** Basic characteristics and bivariate analysis of the influenza vaccination uptake among the elderly with disabilities.

	Total		Non-vaccinated		Vaccinated		p-value
Variable name	N	%	N	%	N	%	
Total	283172	100.00	190577	67.30	92595	32.70	
Disability type							<0.001
Physical	254134	89.75	170917	67.25	83217	32.75	
Mental	22979	8.11	15836	68.92	7143	31.08	
Both	6059	2.14	3824	63.11	2235	36.89	
Disability severity							<0.001
Mild	97106	34.29	59854	61.64	37252	38.36	
Moderate	91217	32.21	62005	67.98	29212	32.02	
Severe	61124	21.59	44693	73.12	16431	26.88	
Very severe	33725	11.91	24025	71.24	9700	28.76	
Gender							<0.001
Male	147161	51.97	99610	67.69	47551	32.31	
Female	136011	48.03	90967	66.88	45044	33.12	
Age							<0.001
65–69 years	58703	20.73	40689	69.31	18014	30.69	
70–74 years	59447	20.99	37566	63.19	21881	36.81	
75–79 years	60229	21.27	36979	61.40	23250	38.60	
80–84 years	51342	18.13	33800	65.83	17542	34.17	
≧85 years	53451	18.88	41543	77.72	11908	22.28	
(Mean ± SD)	77.07±7.86		77.46±8.27		76.26±6.87		<0.001^a^
Education level							<0.001
Illiterate or elementary	196707	69.47	132556	67.39	64151	32.61	
Junior high school	15598	5.51	10125	64.91	5473	35.09	
Senior (vocational) high school	15440	5.45	9991	64.71	5449	35.29	
College or university	11020	3.89	7157	64.95	3863	35.05	
Unknown	44407	15.68	30748	69.24	13659	30.76	
Marital status							<0.001
Single	20138	7.11	13897	69.01	6241	30.99	
Married	178283	62.96	117214	65.75	61069	34.25	
Divorce or widow	8291	2.93	5340	64.41	2951	35.59	
Unknown	76460	27.00	54126	70.79	22334	29.21	
Aborigine							<0.001
No	279131	98.57	187556	67.19	91575	32.81	
Yes	4041	1.43	3021	74.76	1020	25.24	
Premium-based monthly salary (NT$)							<0.001
Dependent	7836	2.77	5340	68.15	2496	31.85	
≦22,800	156491	55.26	106346	67.96	50145	32.04	
22,801–36,300	87390	30.86	58274	66.68	29116	33.32	
≧36,301	31455	11.11	20617	65.54	10838	34.46	
CCI							<0.001
0	87969	31.07	68721	78.12	19248	21.88	
1	57500	20.31	35369	61.51	22131	38.49	
2	50958	18.00	31066	60.96	19892	39.04	
3	34844	12.30	21340	61.24	13504	38.76	
≧4	51901	18.33	34081	65.67	17820	34.33	
OPD during the preceding year							<0.001
<25 times	144722	51.11	114342	79.01	30380	20.99	
≧25 times	138450	48.89	76235	55.06	62215	44.94	
Hospitalization during the preceding year							<0.001
No	188352	66.52	126045	66.92	62307	33.08	
Yes	94820	33.48	64532	68.06	30288	31.94	
Influenza vaccination during the preceding year							<0.001
No	199375	70.41	162407	81.46	36968	18.54	
Yes	83797	29.59	28170	33.62	55627	66.38	
Health examination during the preceding year							<0.001
No	212405	75.01	155606	73.26	56799	26.74	
Yes	70767	24.99	34971	49.42	35796	50.58	
Hospital level							<0.001
Medical center	58864	20.79	39201	66.60	19663	33.40	
Regional hospital	98802	34.89	62019	62.77	36783	37.23	
District hospital	72937	25.76	43088	59.08	29849	40.92	
Clinic	52569	18.56	46269	88.02	6300	11.98	
Hospital ownership							<0.001
Public	63250	22.34	41127	65.02	22123	34.98	
Private	219922	77.66	149450	67.96	70472	32.04	

^a^ t-test

Abbreviation: CCI, Charlson Comorbidity Index; OPD, outpatient department

In Table 2, odds ratios (ORs) and 95% confidence interval (CI) were estimated using logistic regression models to examine the associations between receiving influenza vaccination and the study variables. The results observed in the adjusted model were the net effects of each independent variable after controlling for other variables. The probability of receiving influenza vaccination was significantly associated with health service utilization patterns during the preceding year. The elderly people with disabilities who received influenza vaccination and health examination during the preceding year were more likely to receive influenza vaccination than those who did not receive influenza vaccination and health examination (influenza vaccination: adjusted odds ratio [AOR] = 6.80, 95% confidence interval [CI]: 6.67–6.93; health examination: AOR = 1.66, 95% CI: 1.62–1.69, respectively). The subjects who had more than 25 OPD visits during the previous year were more likely to receive influenza vaccination than those who had fewer OPD visits (AOR = 1.85, 95% CI: 1.81–1.89).

**Table 2:** Logistic regression models for the influenza vaccination uptake among the elderly with disabilities.

	Unadjusted Model			Adjusted Model		
Variable name	OR	95% CI	p-value	AOR	95% CI	p-value
Disability type						
Physical (ref.)	1.00			1.00		
Mental	0.93	(0.90–0.95)	<0.001	0.95	(0.92–0.98)	0.005
Both	1.20	(1.14–1.27)	<0.001	1.15	(1.08–1.22)	<0.001
Disability severity						
Mild (ref.)	1.00			1.00		
Moderate	0.76	(0.74–0.77)	<0.001	0.88	(0.86–0.89)	<0.001
Severe	0.59	(0.58–0.60)	<0.001	0.76	(0.74–0.78)	<0.001
Very severe	0.65	(0.63–0.67)	<0.001	0.81	(0.78–0.84)	<0.001
Gender						
Male (ref.)	1.00			1.00		
Female	1.04	(1.02–1.05)	<0.001	0.99	(0.98–1.01)	0.557
Age						
65–69 years (ref.)	1.00			1.00		
70–74 years	1.32	(1.28–1.35)	<0.001	1.08	(1.05–1.11)	<0.001
75–79 years	1.42	(1.39–1.46)	<0.001	1.17	(1.14–1.20)	<0.001
80–84 years	1.17	(1.14–1.20)	<0.001	1.04	(1.01–1.08)	0.005
≧85 years	0.65	(0.63–0.67)	<0.001	0.77	(0.75–0.80)	<0.001
Education level						
Illiterate or elementary (ref.)	1.00			1.00		
Junior high school	1.12	(1.08–1.16)	<0.001	1.07	(1.03–1.12)	0.001
Senior (vocational) high school	1.13	(1.09–1.17)	<0.001	1.10	(1.05–1.14)	<0.001
College or university	1.12	(1.07–1.16)	<0.001	1.07	(1.02–1.12)	0.007
Unknown	0.92	(0.90–0.94)	<0.001	0.99	(0.96–1.01)	0.258
Marital status						
Single (ref.)	1.00			1.00		
Married	1.16	(1.12–1.20)	<0.001	1.05	(1.01–1.09)	0.020
Divorce or widow	1.23	(1.17–1.30)	<0.001	1.00	(0.93–1.06)	0.869
Unknown	0.92	(0.89–0.95)	<0.001	0.96	(0.93–1.00)	0.072
Aborigine						
No (ref.)	1.00			1.00		
Yes	0.69	(0.64–0.74)	<0.001	0.48	(0.44–0.52)	<0.001
Premium-based monthly salary (NT$)						
Dependent (ref.)	1.00			1.00		
≦22,800	1.01	(0.96–1.06)	0.725	1.04	(0.98–1.10)	0.257
22,801–36,300	1.07	(1.02–1.12)	0.008	1.00	(0.94–1.06)	0.941
≧36,301	1.13	(1.07–1.19)	<0.001	1.01	(0.95–1.08)	0.729
CCI						
0 (ref.)	1.00			1.00		
1	2.23	(2.18–2.29)	<0.001	1.23	(1.91–1.26)	<0.001
2	2.29	(2.23–2.34)	<0.001	1.20	(1.16–1.23)	<0.001
3	2.26	(2.20–2.32)	<0.001	1.16	(1.12–1.20)	<0.001
≧4	1.87	(1.82–1.91)	<0.001	0.98	(0.94–1.01)	0.151
OPD during the preceding year						
<25 times (ref.)	1.00			1.00		
≧25 times	3.07	(3.02–3.12)	<0.001	1.85	(1.81–1.89)	<0.001
Hospitalization during the preceding year						
No (ref.)	1.00			1.00		
Yes	0.95	(0.93–0.97)	<0.001	0.66	(0.65–0.67)	<0.001
Influenza vaccination during the preceding year						
No (ref.)	1.00			1.00		
Yes	8.68	(8.52–8.84)	<0.001	6.80	(6.67–6.93)	<0.001
Health examination during the preceding year						
No (ref.)	1.00			1.00		
Yes	2.80	(2.76–2.85)	<0.001	1.66	(1.62–1.69)	<0.001
Hospital level						
Medical center (ref.)	1.00			1.00		
Regional hospital	1.18	(1.16–1.21)	<0.001	1.06	(1.04–1.09)	<0.001
District hospital	1.38	(1.35–1.41)	<0.001	1.13	(1.10–1.16)	<0.001
Clinic	0.27	(0.26–0.28)	<0.001	0.48	(0.47–0.50)	<0.001
Hospital ownership						
Public (ref.)	1.00			1.00		
Private	0.88	(0.86–0.89)	<0.001	1.09	(1.07–1.12)	<0.001

Abbreviation: CCI, Charlson Comorbidity Index; OPD, outpatient department; OR, odds ratio; AOR, adjusted odds ratio; CI, confidence interval

In addition, the disabled elderly who were afflicted with both physical and mental disabilities (AOR = 1.15, 95% CI: 1.08–1.22), were 70–84 years old (70–74 years: AOR = 1.08, 75–79 years: AOR = 1.17, 80–84 years: AOR = 1.04, respectively), had a higher education level (junior high school: AOR = 1.07, senior high school: AOR = 1.10; college or university: AOR = 1.07, respectively), were married (AOR = 1.05), and had more CCIs (1 CCI: AOR = 1.23, 2 CCI: AOR = 1.20, 3 CCI: AOR = 1.16, respectively) were significantly more likely to be vaccinated.

However, those who had more severe disabilities (moderate: AOR = 0.88, severe: AOR = 0.76, very severe: AOR = 0.81, respectively), were aged 85 years and older (AOR = 0.77, 95% CI: 0.75–0.80), were aborigines (AOR = 0.48, 95% CI: 0.44–0.52), and had been hospitalized during the previous year (AOR = 0.66, 95% CI: 0.65–0.67) were significant less likely to be vaccinated.

## Discussion

To the best of our knowledge, this study is the first to use a nationwide population-based dataset to investigate the seasonal influenza vaccination rate and its associated factors among elderly people with disabilities under a universal health insurance coverage system. This study presents two main findings. First, among the elderly people with disabilities in Taiwan, 32.70% received seasonal influenza vaccination in 2008. Second, the strongest predictor for getting vaccinated in 2008, among older Taiwanese with disabilities, was their experience of receiving an influenza vaccination in the previous year. Frequent OPD use and undergoing health examinations in the previous year also showed a moderate but significant association with receiving an influenza vaccination.

The immunization rate recommended by Healthy People 2010 for the population aged 65 years and older is 90% [28]. However, our results revealed that approximately 32.70% of the elderly people with disabilities were vaccinated in 2008, which is lower than the 44% influenza vaccination coverage rate for the entire general elderly population in Taiwan [29]. Unlike previous studies conducted in the United States [17, 19–22], our study found that elderly people with disabilities were less likely to receive influenza immunization than those without disabilities. Studies on disabilities have varied in the populations studied, and most previous studies have employed different definitions of disability, making it difficult to compare their results [17, 19–22]. In this study, the disability and immunization status were derived from the National Disability Registration System and health services claims dataset from Bureau of NHI, respectively. Those data have been confirmed by the government agency and could prevent recall bias from the subjects. Thus, we are confident that the vaccination rates reported here are considerably close to the reality in Taiwan. Since the seasonal influenza vaccine is free for people aged 65 years and older in Taiwan under the NHI program, it seems that such a low vaccination rate among elderly people with disabilities could not be attributed to financial concerns. Based on the findings from our analysis, a discussion of the factors explored in this study is presented here.

In our study, the experience of receiving vaccination and undergoing health check-up in the previous year turned out to be the strong and more moderate predictors for an influenza vaccination uptake among the elderly people with disabilities, respectively. Both of them are related with health behavior theories which indicated that people who have more positive beliefs and attitudes towards a preventive service are more likely to take actions to utilize the service [17, 30]. In addition, people who seek out health promotion information have an increased health awareness and are more likely to adopt enhanced health-protective behaviors. A systematic review showed that if elderly adults have a positive attitude and sufficient information toward prevention, they are more likely to receive vaccination than those who do not [3]. More importantly, a positive prior experience with the vaccination may play a crucial role to the next vaccination. If disabled elderly or their caregivers have a positive prior vaccination experience, they are more confident in the effectiveness of the vaccine. Although the limited functional status of disabled elderly may decrease the likelihood of vaccine uptake since access might depend on transportation or assistance, once the arrangements are set up, influenza vaccine is likely to become a yearly routine [3].

On the other hand, we also found that disabled elderly with more comorbidities or frequent OPD use during the preceding year had higher influenza vaccination rates. One plausible explanation may be that disabled elderly with more chronic illnesses are likely to develop severe complications once they contract influenza viruses; hence, they are more likely to seek information on seasonal influenza and the ways to prevent it [31, 32]. In addition, elderly people with disabilities and more comorbidities may use more OPD services; thus, they may also have more opportunities to contact health care professionals who might provide additional vaccination advice [22, 33, 34]. Numerous review articles have indicated that recommendations from a physician, nurse, and other medical staff are crucial in increasing the likelihood of receiving influenza vaccination [31, 32, 35–39].

In Taiwan, the elderly people with disabilities, especially those with high levels of disability, were less likely to receive influenza vaccination. This might be because in accordance with Influenza Vaccination Program, vaccination must be administered by trained professionals at licensed medical facilities (e.g., hospitals, clinics, and long-term care facilities) [25]. To receive influenza vaccination, elderly people with higher levels of disability may require extra assistance from family members and other caregivers [3, 22]. If they and their caregivers are unaware of the importance of vaccination, and if the caregivers do not assist them in the processes, they may be unable to receive influenza vaccination. Disability severity might restrict the possibility of receiving vaccination. Therefore, educational and health promotion efforts concerning the potential benefits of influenza vaccination should not be limited to elderly people with disabilities, but should also be extended to their family members and caregivers. Further, providing assistance to people with disabilities for the indirect costs of their vaccination (e.g., special vehicles, extra personnel, time-consuming and additional coordination) can enhance their willingness to receive influenza vaccination [3]. Moreover, elderly people with disabilities have poor mobility and are frail; hence, extra efforts are necessary for them to receive vaccination. Following suggestions from previous researchers, policy makers are recommended to use incentives to encourage vaccine providers to deliver vaccination services to places where elderly people with disabilities live [32, 39, 40] or other convenient locations, such as community pharmacies, supermarkets, or churches [3]. Future intervention programs designed to improve use of influenza vaccination among the elderly with disabilities could consider incorporating some of our findings into their programs.

As regards other significant factors, a large population-based sample was analyzed in this study and, consequently, a high statistical power might cause some weak-association predictors to reach the traditional .05 significant level [41]. We have, accordingly, focused our discussion only on factors with larger odds ratios. We advise readers to interpret the predictors with borderline-odds ratios (e.g., being married, older, or with a higher level of education) in a more conservative way.

This study has some potential limitations. First, the data used in this study were derived from linking the National Disability Registration System and health service claims dataset from the National Health Insurance program in 2008. We could have selected the year 2009 or 2010; however, in order to avoid the pandemic period of H1N1 outbreak in Taiwan, which started in late 2009 and lasted through the early months of 2010, we decided to use the data from 2008, which was the most recent and complete dataset available. When we looked at the percentage distribution of gender and level of disabilities from the National Disability Registration System between 2008 and 2014 and only small changes occurred. More important, no revisions on the policy of providing free influenza vaccinations to the aged 65 and over has been made, so that we believe that the results of this study is still relevant. Second, this study has certain inherent limitations because of the use of the secondary claims data. Although our study has the advantage of including numerous crucial individual characteristics extracted from the Ministry of the Interior and NHI research database, it could not control the bias caused by other unobserved confounding risk factors, such as health behaviors (e.g., smoking and obesity) and beliefs and attitudes toward vaccination (e.g., concerns regarding possible side effects and beliefs in the efficacy of vaccination). It is critical to conduct surveys to understand these beliefs among the elderly with disabilities, so that effective intervention programs can be put in place. Third, this study only analyzed subjects who received free influenza vaccination. There might be a small percentage of people with disabilities in Taiwan who pay out-of-pocket for influenza vaccination. These people are not accounted for in the NHI claims data. Forth, because this study is a cross-sectional study, all the discussions presented here are only epidemiological associations.

## Conclusion

Influenza vaccination among elderly people with disabilities is a critical public health concern. In 2008, only 32.70% elderly people with disabilities in Taiwan received seasonal influenza vaccine, which is lower than the figure for the entire general elderly population. Our findings suggest that free vaccination alone is not enough to substantially raise the vaccination rates in this subgroup. To improve the influenza vaccination rates among elderly people with disabilities, an integrated policy tailored to the specific needs of the elderly with disabilities is needed. For example, based on our findings, outreach programs targeting the disabled elderly who have not before received influenza vaccination, have not undergo health check-up, or have fewer contacts with health providers should be implemented. Other programs, such as giving incentives to health providers to deliver the vaccination services to convenient locations for the disabled elderly, reimbursing the indirect costs of getting vaccinated to their caregivers, and disseminating the availability and benefits of being vaccinated through media campaigns should be able to substantially increase the vaccination uptake. Finally, more cost benefits and cost effectiveness analyses of influenza vaccination are needed in order to have a more thorough understanding and delivery services.
